# From pigeons to person: Reimagining social history

**DOI:** 10.1111/tct.13486

**Published:** 2022-03-28

**Authors:** Grainne P. Kearney, Richard L. Conn, Helen Reid

**Affiliations:** ^1^ Centre for Medical Education Queen's University Belfast Belfast UK

1

History taking frameworks, diligently instilled into health professions learners the world over, follow a standardised pattern. ‘Social history’ comes near the end, after exploring the reason for consulting, past medical, family, and medication history. Yet experienced clinicians recognise that a person's social context is fundamental to any consultation. Could reimagining how we teach ‘social history’ within consultation frameworks help reinstate the centrality of the person in healthcare?

Could reimagining how we teach ‘social history’ within consultation frameworks help reinstate the centrality of the person in healthcare?

Questions in a social history are intended to give an overview of a person's situation, lifestyle and habits. The utterance of traditional social history questions often signals a consultation winding down: “Are you working? Do you smoke? Did you ever smoke?” (*vital* to catch out the ex‐smokers); the ubiquitous, “Do you keep pigeons?”, lest a rare fibrotic respiratory condition be missed.

We argue that this fact‐finding approach is deeply problematic. As clinicians, we have learnt through experience that the social history's long‐established questions, while important, are the tip of the iceberg when considering what makes a person an individual. Recognising this, Srivastava (2011) has argued for modernisation of the social history, asking questions that account for changing relationships and social supports beyond traditional family units [1]. The way we position ‘social history’ risks rendering it a tokenistic adjunct to more clinically oriented aspects of the consultation such as ‘history of presenting complaint’. It reduces important details of people's lives to superficial tick‐boxes, compartmentalising vital information about the complex whole. It discourages the flexibility that enables students to explore and respond authentically to sensitive revelations as they emerge. Evidencing the negative consequences of this traditional approach, we see students quickly rhyming off some social history questions for a few easy OSCE (Objective Structured Clinical Examination) marks. This is all the better if they have enough time to calculate smoking ‘pack years’ while simultaneously sanitising hands as they exit the station.

The way we position ‘social history’ risks rendering it a tokenistic adjunct to more clinically oriented aspects of the consultation.

Srivastava invites us to consider that “every patient is a person and illness occurs in the context of multi‐faceted lives” [1]. Patients themselves confirm this; when asked what makes a doctor ‘caring’, a patient replied, ‘He knows everything about it [the illness] but he also knows about me’. [2] Every patient's unique context deserves attention throughout their entire interaction with a health professional. While experienced clinicians may naturally adapt their consulting approach to capture this, we propose that it is now time that we reconsider our approach to history taking so that the version of practice we teach mirrors the practice we aspire to. We propose that, rather than being confined to the ‘social history’, a person's context should be a central strand that winds through a consultation. Recognising that students have to start somewhere and that history taking frameworks are a vital developmental scaffold, we suggest that students should be encouraged to explore relevant person‐specific details within other consultation components. For example, the time to ask about impacts of presenting issues on the person's home life and perhaps employment would be alongside the traditional ‘History of Presenting Complaint’. When asking about ‘Past Medical History’ could be an instructive time to get the patient's view on how this compares with previous illness experience they may have, as well as clarifying alcohol and smoking status if relevant risk factors. A more natural time to inquire about a patient's caring responsibilities would be when asking about family history (Figure 1). This may also be the opportunity to explore patients' concerns that their symptoms may be their presentation of a condition that has affected other members of their family.

**FIGURE 1:**
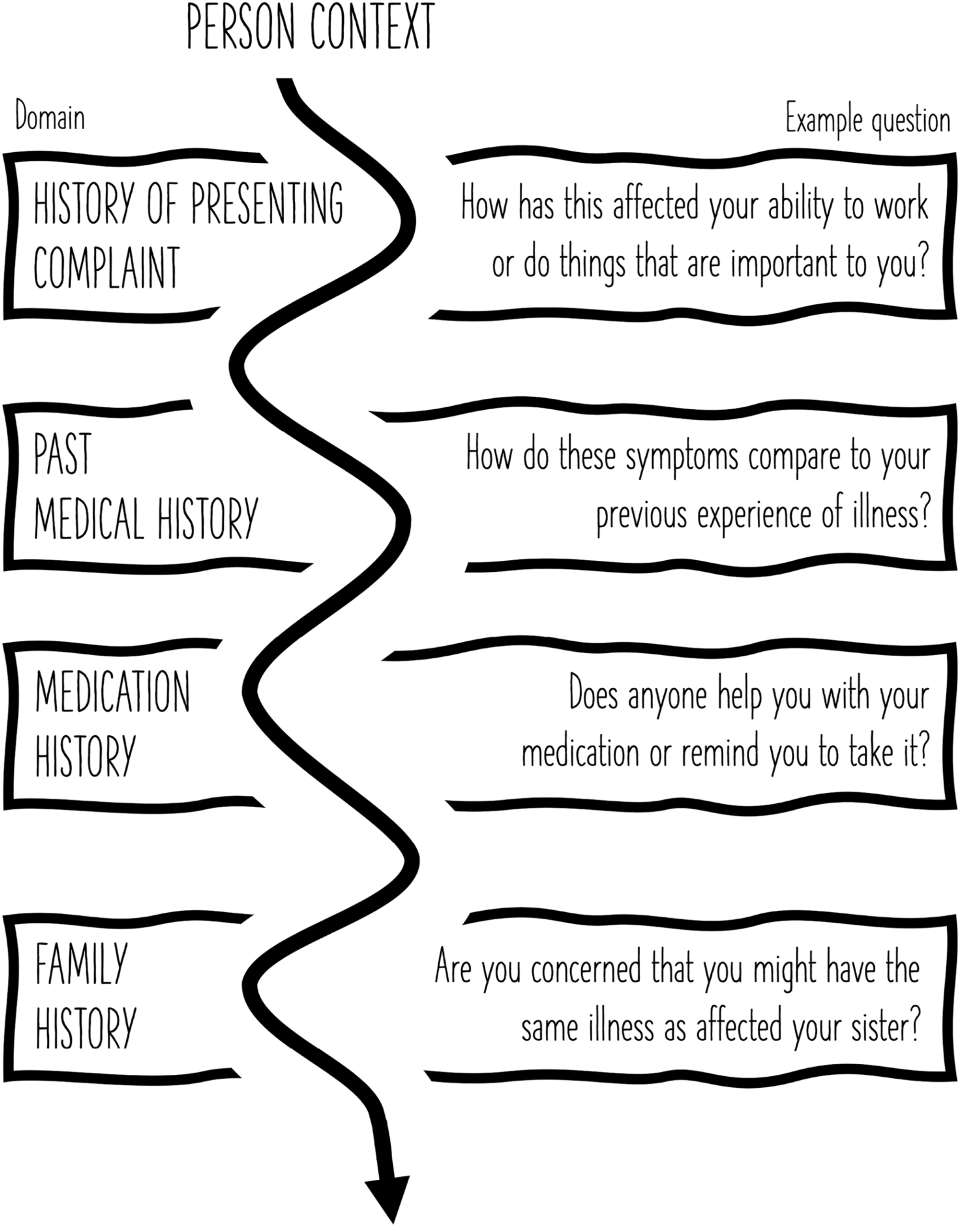
Centralising person context in history taking frameworks

We propose that, rather than being confined to the ‘social history’, a person's context should be a central strand that winds through a consultation.

Implementing this approach with health professions students will necessitate a shift in how we as their educators structure their teaching of consultations, starting with ‘person context’, relating other aspects of the consultation framework to person context throughout and revisiting it at the end again. We have already introduced this idea of ‘person context’ centrality into students' early clinical contact with patients, whereby they structure their reflective report based on the person and their experience of their illness rather than following a disease focused structure. In our teaching around consulting in primary care, we consider the broader context of why a patient presents at that particular moment in time, considering factors such as family dynamics, previous personal or family illness experience and individual work demands. Finally, we ourselves model this approach when students are observing our consultations and use this as a framework for giving feedback to students on their consultation skills. Students have been receptive of this approach, which sits alongside their growing understanding of patients as people. We suggest that for educators interested in advocating for person centredness, this ‘reimagination’ of ‘social history teaching’ may be more akin to a reawakening, sitting comfortable alongside their person‐centred clinical experience.

While this less structured approach might initially challenge students, we anticipate that exploring patients' individual contexts in an integrated way is closer to what they will encounter in practice. As students become more accustomed to actively listening to patients, it will become more natural to explore person context information at relevant stages through the consultation, rather than waiting to ask it all at the end. Recognising that assessments drive learning, educators could promote this approach by abandoning stand‐alone ‘social history’ marks in OSCEs. Instead, more emphasis could be placed on a student's understanding of patient context through global marks or within other history components.

In conclusion, a more person‐centred approach to history taking is to discuss person context throughout a consultation. This approach offers potential to move beyond fact‐finding towards understanding the person's lifeworld. As well as smoothing students' transition to the realities of practice, this could help develop foundations of therapeutic relationships, as a basis for genuine shared‐decision making. The time is now to centralise the person within patient–clinician consultations. And as for those pigeons? Entirely irrelevant in all but a tiny minority of consultations.

## FUNDING INFORMATION

Not applicable.

## CONFLICT OF INTEREST

The authors have no competing interests to declare.

## ETHICS STATEMENT

Ethical approval not required.
